# Economic-Environmental Law Guarantee of the Green and Sustainable Development: Role of Health Expenditure and Innovation

**DOI:** 10.3389/fpubh.2022.910643

**Published:** 2022-06-06

**Authors:** Lin Yang

**Affiliations:** School of Law, Hunan University, Changsha, China

**Keywords:** environmental quality, economic environmental regulations, government health expenditure, innovation, Porter Hypothesis

## Abstract

Environmental regulation is a tool for teaching social and fiscal development that is carbon neutral. The highly polluting food industry in China is a threat to the country's long-term environmental stability and affects public health in a significant way. Therefore, this study investigates the effect of environmental parameters on environmental quality in China's food industry using the cross-sectionally augmented ARDL (CS-ARDL) model over the period of 2010 to 2019. We find that environmental regulations negatively and significantly impact environmental quality. The U-shape relationship exists between environmental regulation and environmental quality. Moreover, government expenditure on health and technological innovation reduces carbon emissions. The study's findings suggest new policy implications supporting the Porter Hypothesis. Finally, this paper offers policy suggestions for China's food industry to enhance its environmental performance.

## Introduction

In China, energy consumption and CO_2_ emissions rank first and second globally. According to the international energy agency, China's primary energy utilization reported 24.27 % of global energy utilization in 2019 (1). Moreover, it was responsible for 28% of the world's total emissions in the same year, resulting in 9825.80 million metric tons of CO2 (2–4). Excessive energy use in China has brought environmental degradation. The conflict between environmental protection and sustainable economic growth makes it difficult to build a harmonious society.

**Figure 1:**
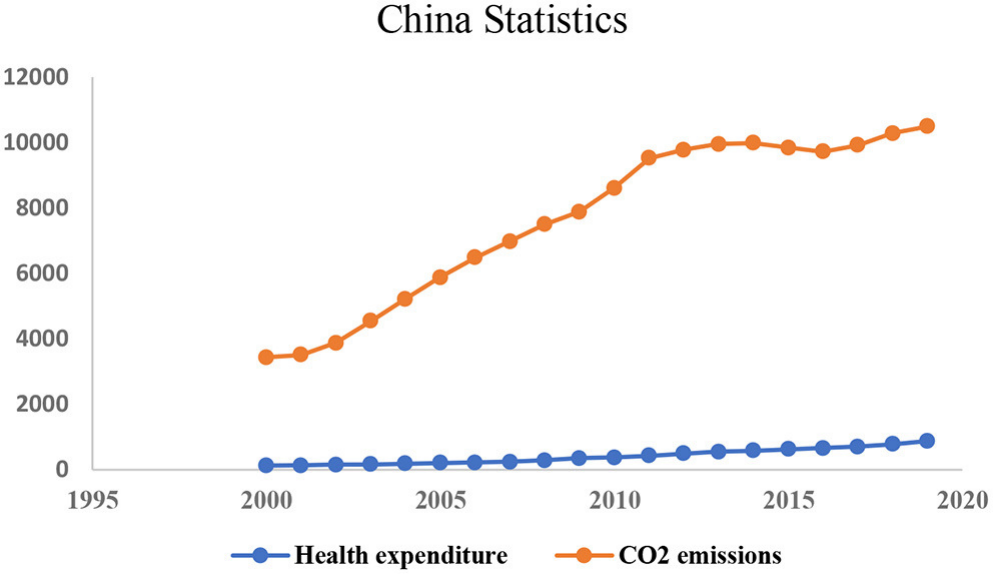
China's health expenditure and CO2 emissions over the period of 2000-to 2019.

Moreover, the degradation of the environment and environmental scientists and politicians are increasingly concerned about carbon dioxide emissions (CO2). As a result of their desire to achieve rapid output growth, many developing and developed countries compromise air quality and environmental health (5, 6). As a result of deteriorating air quality and environmental conditions, Health-related expenses are in greater demand to maintain a healthy lifestyle (7). Seven million people worldwide die prematurely each year because of air pollution. Increasing levels of environmental pollution caused by anthropogenic discharges such as CO_2_ have an impact on the cost of health care spending (8). Health expenditures are constantly rising due to the need for governments to fund a better system of health care delivery and access to public insurance (see Figure 1). The need for health care foundations and insurance is growing as a result of urbanization, industrial expansion, an increase in energy utilization, the development of infrastructure, and the migration of people from rural to urban areas (9). An advanced level of health expenses is indicative of two things: (a) a society that is concerned about public health and individual; and (b) chemicals, air pollution, and harmful diet may come from a high amount of industrial output (10). As a result, examining the interactions between the environment, healthcare spending, and economic growth becomes increasingly essential.

The food industry had a significant adverse effect on the environment due to its importance to national economic growth. The food industry does not consume a lot of energy or emit a lot of emissions. But to meet the demands of 1.4 billion people the industry has evolved and experienced rapid growth over the last few years (11–13). As a result, the food industry in China continues to be a significant consumer of energy, with 57.95 million tons of coal equivalent (tce) of energy, accounting for 2.84 % of China's total industrial energy consumption (14–16). As a result, the issue of carbon emissions caused by industry should be given the attention it deserves. Carbon emission reduction can only be achieved by analyzing the characteristics and primary driving forces of carbon emission in the industry, quantifying the impact of each factor, and proposing specific emission reduction strategies.

The amount of carbon dioxide emitted by industry is highly related to the process by which the industry produces its products (17, 18). In contrast, from the perspective of the industry chain, the relationship between other sectors in the economy will also have an impact on the production activities of the industry, as well as its energy consumption and carbon emission in all aspects of its operation (19–21). It is essential to investigate environmental problems to understand the relationship between the food industry and other industries. To address environmental issues, China has implemented a variety of strategies, ranging from directorial procedures to a more inclusive use of decree, economics, and scientific technology. The construction of China's environmental safety system, which encourages a stern ecological protection and power system, has made significant progress in this process. According to the Kyoto Protocol and Paris Agreement, China actively engages in global environmental governance through multilateral environmental negotiations.

With four discs of central environmental safety reviews since 2016, China has exerted considerable effort to ensure that all industries, including the food industry, are subject to strict macro-controls and environmental governance. New environmental protection laws and environmental tax laws implemented in 2015 and 2018 will put the food industry at risk of being shut down if they don't meet environmental protection and production standards. Some food companies are losing market due to rising financing in environmental safety organizations (22, 23). As a result of implementing local and industry-specific strategies, environmental authority is stronger than ever. When it comes to environmental regulations, food companies are explicitly mentioned in the Work Plan for Prevention and Control of Air Pollution and the Special Action Work Plan for Cleaning and Rectification of Illegal Projects in the Food Industry. Environmental regulation is widely regarded as a valuable tool for reducing pollution (24). Efforts to address environmental pollution's external costs have been successful. The food industry is subject to a wide range of regulations aimed at reducing energy consumption and emissions from the Chinese government (25).

This article aims to investigate the impact of environmental regulations and health expenditure on CO_2_ emissions in China. To be more specific, this research is groundbreaking in three ways: The following differences were observed: (I)As opposed to prior studies, this analysis used different proxies for public and private health expenditures rather than aggregate health expenditures.; (ii) For the first time, CO_2_ emissions were used as a dependent variable, and the EKC framework was used to examine the relationship between environmental pollution, health care spending, and environmental quality; and (iii) Environmental restrictions, health expenditures, and environmental quality were analyzed using quantile regression. The rest of the paper follows this structure. The second part of the paper reviews the relevant literature on competitiveness and environmental regulation. The third section's theoretical underpinnings and methodology are laid out here. Specifying the data and variables for the econometric model is done in the fourth sector. The fifth section presents the study's results and robustness tests. Conclusions and recommendations are presented in the concluding section.

## Literature Review

### Environmental Regulation and Environmental Quality

There has long been research on the connection between business competitiveness and environmental regulation in academia. The compliant Price Hypothesis (26) and the Porter Hypothesis are two of the most commonly studied theories on the consequence of environmental regulation (27–30).

Regulation types and business responses are two variables that have been difficult to pin down but are crucial in determining whether and under what situations environmental regulation has a negative or positive impact on competitiveness (6, 31–34). When it comes to determining how regulation affects competition, the style of instruction may even be more critical than the degree of rigor. Additionally, the association between environmental strategies and the environmental performance and competitiveness of specific industries and businesses may differ. To understand the connection between environmental regulation and environmental quality, it may be necessary to consider these factors in the methods of assessment.

Neoclassicfinancesgrips that the cost of compliance is an important consideration. According to this theory, the cost of environmental protection rises as a result of environmental regulations. A lack of capital investment in technological innovation will lead to a decrease in production efficiency as a result of additional costs. The costs of pollution control are imposed on businesses by environmental regulations. Higher expenses will affect industry investment decisions, productivity, and profitability (35). The compliance Cost Hypothesis was previously supported by previous studies (36). A number of recent studies have also backed up this theory. The Clean Air Act amendments had a significant impact on the efficiency of generator units, according to (37). This regulation, according to him, had a negative impact on more than 90% of coal-fired power plants. According to Zhang (38), environmental regulations hurt pollution-intensive businesses' ability to compete.OECD countries' industrial sectors were studied by Zhang et al. (39). It was recognized that environmental regulation strategies decreased the efficiency of unproductive organizations and pushed them out of the marketplace.

For environmental regulation, the Porter Hypothesis presented a new avenue of investigation. According to Zhang et al. (39), corporations would be forced to innovate and upgrade their industrial structure as a result of environmental regulation. Complying with the law is likely to have a negative impact on innovation compensation. Because of advancements in technology, businesses will be able to lower their environmental pollution treatment costs in the long run. Companies' competitiveness can be improved as a result of environmental regulation by enhancing production efficiency (40). Porter hypothesizes that environmental regulation has an impact on a company's ability to compete and make money, among other things (41). Scholars widely accepted Porter's Hypothesis in the past (42). Recent research has bolstered this hypothesis (43). It originates that environmental regulation in India's cement industry reduces pollution levels and increases energy efficiency, and direct regulations positively affect the construction industry's commercial (44). Market-based green development efficiency and environmental regulation were examined using the DID model (45). Their findings confirmed the Porter Hypothesis by showing that China's carbon emissions interchange structure enhanced green growth efficacy in preliminary provinces. As further evidence of the Porter Hypothesis's validity (46). Environmental regulations, such as green taxes, had a different but optimistic impact on the workforce and yield development in 18 OECD countries.

However, the cogency of the Porter Hypothesis was questioned by some academics. Using data from German firms in 2009 (46) confirmed that the robust Porter Hypothesis was not always legal. According to Bashir et al. (47), 28 subdivisions of China's manufacturing were categorized into efficiency-based groups. From 2003 to 2013, environmental regulations were ineffective in promoting ecological efficiency. Using the DID method (48) investigated the impact of China's advanced environmental protection rule on the productivity of recorded companies. According to their study, the new legislation did not meet expectations in terms of environmental and economic outcomes. The Porter Hypothesis was put to the test by Qu et al. (49), who built a monopolistic competition model. According to the researchers, more capable firms in the same industry benefited from environmental regulations, but less capable ones did not. This is due to the fact that each company's innovation investment strategy is unique.

### Health Expenditure and Environmental Quality

As a final point of reference, the relationship between health expenditure and environmental variables has focused on the second spectrum of research. Compared to the EKC literature, Scientists and researchers have paid little attention to this region of the electromagnetic spectrum. Coscieme et al. (50) led a study on the connection between healthcare costs and CO_2_ emissions. Their study, which was conducted using the STIRPAT framework, discovered that healthcare spending in China was associated with increased CO2 emissions. It was determined that there is a two-way causal relationship between health care spending and CO2 emissions. For example, Popp (51) discovered a reversed U shape relationship between capital income and per health expenditure. There was a view that economic maturity is necessary for disease reduction, developed in the last stages of economic growth. Their findings supported the hypothesis that there is a direct relationship between CO_2_ emissions and health expenditure per capita. Alimi et al. (52) investigated the relationship between carbon dioxide emissions and health expenditure. They discovered that environmental degradation increases health expenditures by analyzing data from 15 West African countries from 1995 to 2014. These findings were found only for community health costs; however, results for private health expenditures were statistically insignificant. The study asserted that individuals are unlikely to spend their own money on health-related issues caused by increased carbon emissions. According to Yahaya et al. (53), using a panel data set consisting of 125 emerging countries, CO_2_, nitrous oxide, and money per capita all have a favorable long-term impact on health expenses. But in the short term, Healthcare costs per person were unaffected by increases in nitrous oxide and Sulfur dioxide emissions in emerging nations. An additional finding of recent research by Ibukun and Osinubi (54) was that improved health expenditures result from poorer air and water quality.

According to the findings, greenhouse gas (GHG) emissions are healthcare costs that a number of factors can predict. CO_2_ is the most significant contributor to global warming among all greenhouse gases, carbon monoxide was then released (55). Paramati et al. (56) examined group data from 125 countries and discovered that greenhouse gas emissions significantly increase healthcare expenditures. Khan et al. (57) extends this model further As additional determinants of health, population density, and infant mortality are included. According to Alhajeri et al. (58), a fading environment has a negative impact on health indicators. According to the findings of a study conducted in Nigeria, time-series data from the ARDL model, population density, and infant mortality positively affect healthcare spending. Furthermore, greenhouse gas emissions have a negative impact on health care expenses. Peng (59) discovered that greenhouse gases (GHG) are harmful to human health in Nigeria. On the other hand, the findings were at odds with other country-specific studies; for example, Qudrat-Ullah and Nevo (60) discovered an affirmative relationship between greenhouse gas emissions and Malaysia's healthcare expenditures.

It has been discovered that health expenditures significantly contribute to carbon emissions. For example, according to Yu and Wang (61), expenditure on health care has a positive correlation with levels of CO2 emissions. Kshetri (62) discovered similar results for the Middle East and North Africa (MENA) region. Their research found experimental data of an expansion in environmental destruction as a result of an increase in healthcare spending. Burns (63) presented findings supporting previous evidence that health expenditure is a significant contributor to carbon emissions, based on a panel of 20 countries. Zaman and Abd-el Moemen (64) provided findings from their study of the relationship between electricity production and health expenditures, which revealed that health expenditures contribute to the depletion of the environment. Fiodor et al. (65) discovered that health outlays increase carbon emissions by GMM. FMOLS approaches are being used to analyze data from 58 nations participating in the Belt and Road Initiative (65) use the ARDL co-integration model to examine the relationships between health expenditure, CO_2_ emissions, and GDP per capita in 18 OECD countries over the period 1975–2017. In the case of New Zealand and Norway, they discovered bidirectional interconnection between health expenditure and CO_2_ emissions.

The results of various econometric methods used to investigate the relationship between CO_2_ and health expenditure may or may not be affected. For example, Idrees and Majeed (66) used 2SLS and 3SLS to analyze Pakistani health expenditure data from 1998 to 2017 and discovered that CO_2_ positively affects health expenditures. Their findings are similar to those of Sarkodie and Strezov (67), who used FMOLS and DOLS data from 1995 to 2017 for Pakistan to arrive at their conclusions. A similar conclusion was reached for China by Sulich and Sołoducho-Pelc (68) and Yu and Wang (61), who discovered that the exact Health care costs are inflated by the use of waste gas and garbage. Data from China was used in both investigations at the province level and used two different techniques, FMOLS and quantile regression models, to analyze the data. However, according to Wang et al. (69), quintile regression appears to be better for regional comparisons based on income since the study's objectives are to be met. Their province-level study discovered that the results for low-income regions were different from the results for medium- and upper-income regions.

## Methodology and Data

### Econometric Model Setting

#### CD Test

Specifically, The homogeneity test devised by Su and Urban (70) is used in this study, and the cross-sectional dependency (CD) test developed by Hou et al. (71) as part of its estimation strategy (2004). The examination of CD and heterogeneity has emerged as a severe issue in panel data analysis because CD and heterogeneity may produce incorrect or misleading results (72). As a result, classifying dependency in panel data is critical because of the expansion of socio-economic systems. There is the potential for cross-sectional reliance in the event of arbitrary shared shocks. This research uses a more sophisticated CD test (73).

#### Unit Root Test

Pesaran (74) developed the cross-sectional integration properties of variables (CIPS) unit root test, which we use to examine the variables' integration properties. In order to avoid bias in estimates, the adoption of an appropriate unit root test is essential that takes into account the CD. Many first-generation unit root tests make assumptions about cross-sectional independence, which can lead to incorrect estimates. When it comes to identifying the existence of CD, the use of CIPS is very common. The following is the equation for the test:

#### Co-integration Tests

Co-integration relationships between the underlying variables must be identified after the stationarity diagnostics. Because of the heterogeneity and CD, Westerlund's second-generation co-integration test is the most appropriate (2007). There is no restriction on common factors in this test. Because of this, it is preferable to prior generation co-integration tests, such as those b (75).

### Cross-Sectionally Augmented ARDL (CS-ARDL)

The presence of cross-sectional heterogeneity in a panel data model with large N and T is suggested by the large N and T (76). Because of heterogeneity and cross-section dependence, traditional approaches such as first difference estimation are not appropriate. The generalized method of moments (GMM), random effects, and fixed effects are not permitted (77, 78). This study will use a recently developed approach known as the cross-sectionally augmented autoregressive distributed lags model (CS-ARDL) to obtain short-run and long-run estimate results (79). The following is the basic model for CS-ARDL obtained by transforming Equation (1):


(1) $$\begin{array}{r} y_{it}=\vartheta_{i}+\overset{p}{\underset{l=1}{\sum }}\gamma_{il}y_{i,t-l}+\overset{q}{\underset{l=0}{\sum }}\theta_{il}^{{}^{\prime }}X_{i,t-l}+\varepsilon_{it} \end{array}$$

This method is appropriate for use when there is a concern about heterogeneity, as it is robust to omitted variables, endogeneity, non-stationarity, and cross-sectional dependence, among other characteristics (80, 81). This approach proposes that the conventional ARDL approach be supplemented by including their lags, dependent variable, and cross-section averages of covariates in addition to the traditional ARDL approach.

Specifically, the cross-sectional averages for the covariates and the dependent variable are contained in Equation (3). Furthermore, the lag length for the cross-section averages is denoted by q, and the error term is denoted by ε_*it*_. The unobserved common factor, which causes dependency among cross-sectional units, is denoted by *f*_*t*_. Detrended cross-sectional averages deal with the common factors, and their lags are represented by the Equation (3). The pooled mean group (PMG) approach will estimate the coefficients in Equation (2).


(2) $$\begin{array}{r} y_{it}=\vartheta_{i}+\sum_{l=1}^{p}+\sum_{l=0}^{q}\theta_{il}^{{}^{\prime }}X_{i,t-l}+\sum_{l=0}^{q}\delta_{i,l}^{{}^{\prime }}\bar{Z_{t-l}}+\varepsilon_{it}\\ \;\bar{Z_{t}}=\left(\bar{y_{t}},\bar{X_{t}^{{}^{\prime }}}\right)\\ \;\varepsilon_{it}=\pi_{i}^{{}^{\prime }}f_{t}+\mu_{it} \end{array}$$

It is possible to compute the long-run coefficients using Equation (3), which is given below as follows:


(3) $${\hat{\pi }}_{i}=\;\frac{\sum_{l=0}^{q}{\hat{\theta^{\prime }}}_{il}}{\sum_{l=1}^{p}{\hat{\gamma }}_{il}}$$

This model's error correction (ECM) form can be obtained by transforming Equation (1) (82). The ECM model is defined as follows in Equation (4) below:


(4) $$\begin{array}{r} y_{it}=\vartheta_{i}\left[y_{i,t-1}-\emptyset_{i}X_{it}\right]-\overset{p}{\underset{l=1}{\sum }}\gamma_{il}y_{i,t-l}+\overset{q}{\underset{l=0}{\sum }}\theta_{il}^{{}^{\prime }}X_{i,t-l}\\ +\overset{q}{\underset{l=0}{\sum }}\delta_{i,l}^{{}^{\prime }}\bar{Z_{t-l}}+\varepsilon_{it} \end{array}$$

Where,


$$\begin{array}{l} \hat{\vartheta_{i}}=-\left(1-\overset{p}{\underset{l=1}{\sum }}{\hat{\gamma }}_{il}\right)\\ \;{\hat{\emptyset }}_{i}=\frac{\sum_{l=0}^{q}{\hat{\theta }}_{il}^{{}^{\prime }}}{\hat{\vartheta_{i}}} \end{array}$$

**Figure 2:**
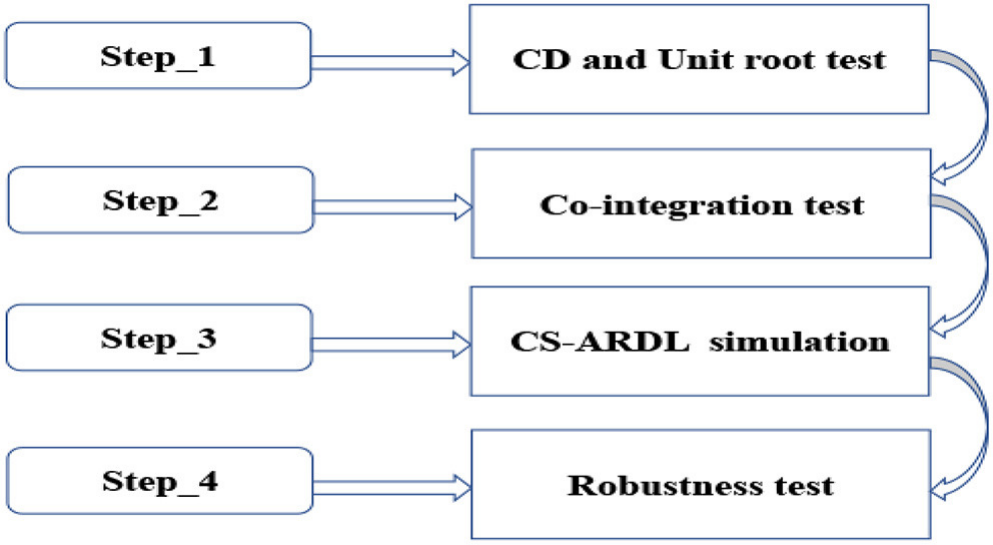
Methodological frame work.

As illustrated in Figure 2, this study followed several standard phases of panel data analysis, as indicated by the arrows.

### Variables and Data Sources

#### Dependent Variable

According to the China Energy Statistical Yearbook, the food industry uses five types of final energy: coal, petroleum products, natural gas, heat, and electricity. This includes four sub-industries: food processing, food manufacturing, beverage manufacturing, and tobacco manufacturing. The amount of CO_2_ emitted by each industry isn't readily apparent. The IPCC methodology is used to estimate CO_2_ emissions from the food industry in 29 Chinese provinces based on energy consumption. In order to calculate CO_2_ emissions, a variety of energy sources must be taken into account. The food industry's CO_2_ emissions from 2010 to 2019 are calculated in this paper by adding up all of the products made with the various types of energy used and their corresponding CO_2_ emission factors.

#### Core Explanatory Variable

We have environmental regulation and government health expenditures among the most important explanatory variables. In reality, the food industry does not have a standardized environmental regulation tool. Because the industrial sector is regarded as the primary source of environmental pollution, this paper represents environmental regulation in the food industry by examining regional initiatives. This paper categorizes environmental regulation instruments into three categories based on their implementers: command-based environmental regulation, market-based environmental regulation, and public-based environmental regulation. Command-based environmental regulation is the most common type of environmental regulation. When environmental regulations are developed with public participation in mind, it highlights the importance of citizens. To determine the intensity of environmental regulation in each province, it is necessary to compare the number of environmental petitions filed in each province to the total number of environmental petitions. Table 1 presents the descriptive statistics of each study variable.

**Table 1:** Descriptive statics.

**Variable**	**Variable description**	**Mean**	**Std. dev**.	**Min**	**Max**
CO_2_	CO_2_ emissions in (million tons)	281.164	227.653	7.883	1168.105
ER	Public-based regulation	0.373	0.295	0.000	1.719
HEXP	Government health expenditure	409.73	237.34	128.20	880.19
Innovation	Technical innovation	16.387	29.478	0.069	195.828
Structure	Industrial structure	0.485	0.075	0.200	0.620
TO	Foreign direct investment	131.975	165.562	0.188	980.703
CS	Capital structure	7.061	10.369	0.003	54.157
ES	Energy structure	0.072	0.056	0.001	0.324

#### Control Variable

##### Technology Innovation

It has been widely accepted that technological innovation is an effective means of reducing CO_2_ emissions in recent years, as evidenced by a number of academic studies. R&D investment is a sure bet for developing new ideas that can stand independently. Research and development investment increases the level of independent innovation. So, investment in R&D is a measure of the level of independent innovation.

##### Trade Openness

The quality of the environment may be affected differently if the trade is more open. Positively, FDI aids in the growth of the industrial sector. The host country's technological and management skills can be continually improved as a result of the spillover effects, resulting in a green transformation of the industry. A drawback of industrial transfer is that some of the most polluting and energy-intensive industries in developed countries are brought to the countries where they will be used. Malek and Desai (83) divided the environmental impact of trade into three categories: scale, structural, and technological. Stopper et al. (84) used this framework to explain how foreign direct investment (FDI) affects energy intensity. When we convert foreign direct investment (FDI) into RMB. The fixed asset price index (the year 2000 = 100) is used to adjust the average exchange rate.

##### Industrial Structure

Structure changes have the potential to have an impact on environmental quality. The service sector has a lower energy intensity than the agricultural and industrial sectors, which significantly improves over the former. Because of the industry's upgrading and restructuring, the proportion of energy-intensive sub-sectors is decreasing, while the proportion of technology-intensive sub-sectors is increasing. It will result in a reduction in the energy intensity of the manufacturing industry. As a result of the dominance of secondary industries in China, there is an increase in energy consumption as well as pollution emissions. Consequently, the ratio of secondary industry output value to GDP is used in this paper as a proxy for the industrial structure of the economy.

##### Energy Structure

Restructuring and optimizing the energy structure is essential to environmental improvement. When environmental pollution is considered, the energy structure significantly impacts economic efficiency and performance. Gondal et al. (85) discovered that a disproportionate reliance on fossil fuels caused a lack of energy efficiency. Coal consumption in the food industry as a percentage of total energy consumption is used to represent energy structure in this research.

## Empirical Results and Discussion

### CD and Unit Root Test

According to the panel data analysis, the majority of environmental economists pay close attention to the concept of complementarity. By ignoring the CD that has been revealed by all of the tests that we have conducted, the results become unreliable (86). In Table 2, the statistical explanations for cross-sectional dependence, rejection, and the presence of confirmation of CD are provided (LM test). This demonstrates how an increase in one province directly affects another province.

**Table 2:** Results of CD and LM test.

**Variable**	**LM**	**CD**
CO_2_	39.368***	13.300***
ER	13.120***	10.042***
HEXP	60.230***	19.988***
Innovation	67.593***	3.420***
Structure	73.131***	14.725***
TO	26.800***	0.188***
CS	15.742***	10.450***
ES	31.048***	6.398***

***1% significance level.

The results of the unit root test conducted by Pesaran (74) are presented in Table 3. As a result of the findings, the initial difference, or I(1), is the point at which all variables are stationary. It is possible to acquire both short-term and long-term findings in this study using CS-ARDL and subsequent co-integration.

**Table 3:** Unit root test.

**Variables**	**Level**		**First difference**		
	**Constant**	**Constant and trend**	**Constant**	**Constant and trend**	**Integration order**
CO_2_	−2.035	−2.109	−3.558***	−3.750***	I(1)
ER	−2.038	−2.096	−4.634***	−4.641***	I(1)
HEXP	−1.928	−2.151	−3.992***	−4.311***	I(1)
Innovation	−1.317	−1.736	−3.450***	−3.597***	I(1)
Structure	−2.057	−2.067	−4.784***	−4.807***	I(1)
TO	−1.809	−2.236	−4.148***	−4.235***	I(1)
CS	−1.458	−2.361	−3.032***	−3.591***	I(1)
ES	−1.519	−1.625	−3.340***	−3.417***	I(1)

^***^represent 1, 5, and 10% significance levels.

The results of the error correction mechanism co-integration experiment conducted by Westerlund (87) are shown in Table 4. The findings indicate that model-1, model-2, and model-3 have a long-term cointegrating relationship.

**Table 4:** Westerlund's (87) ECM-based approach.

**Model**	**Gt**	**Ga**	**Pt**	**Pa**
1	−4.085***	−16.193***	−35.751***	−4.085***
2	−4.157***	−17.834***	−31.972***	−4.157***
3	−3.715***	−17.933***	−28.444***	−3.715***

1, 5, and 10% significance levels represents the ^***^respectively.

### CS-ARDL Results

Table 5 shows the results of the cross-sectionally augmented ARDL long-run and short-run simulations for the error correction term (ECM). The ECM indicates the rate at which the adjustment or correction is made to achieve equilibrium. If the ECM is negative and statistically significant, the value of the term shows convergence, and if it is positive and statistically significant, the value shows divergence. The calculated value is 0.634, which is statistically significant, and it indicates that a 68 % adjustment occurs almost every year. In other words, the rate at which the disequilibrium is corrected and the rate at which the equilibrium is reached is 63.4%. Following the f-statistics value, the model is highly statistically significant, and it also has a lower root mean squared error (RMSE) value than other models.

**Table 5:** Augmented mean group.

**Variables**	**Short-run**	**Long-run**
ER	−0.13***[2.44]	−0.080***[2.35]
	(0.015)	(0.019)
HEXP	−0.57***[4.32]	−0.34*** [4.60]
	(0.000)	(0.002)
Innovation	−0.415***[−1.87]	−0.262***[−1.94]
	(−0.061)	(−0.052)
Structure	0.051**[2.19]	0.030**[2.19]
	(0.028)	(0.029)
TO	0.33***[1.64]	−0.820*[2.75]
	(0.015)	(−0.019)
CS	0.44***[2.52]	−0.64*[2.65]
	(0.001)	0.001
ES	0.41***[−1.87]	0.0026***[−1.94]
	−0.061	−0.052
ECM(−1)	−0.634***[−13.91]	–
	0.002	
CD-statistics		1.69 (0.0917)
RMSE		0.01
F-statistics	2.05*** (0.000)

1, 5, and 10% significance levels represents the ^***^, ^**^, and ^*^ respectively.

In this sample, environmental regulation, represented by the variable ER, reduces emissions by 0.1186% over the long term. Environmental regulations that are more stringent and have a greater number of regulatory policies are more effective at lowering pollution levels. Companies' temporary response to regulation increases the cost of treating wastewater and exhaust gases. It's a waste of money because it competes with the original production investments and doesn't improve energy and environmental efficiency (88). This means that companies will take into account their long-term efforts to reduce emissions as ER strengthens. The technology for reducing pollution reserves will devote more time and resources to research to improve energy and environmental performance. The administration may have an undeveloped managing system in the early stages of CER, which is known as command-based regulation. When governments invest in projects aimed at reducing pollution, they adhere to the same set of standards. This means that all businesses' energy and environmental performance must be worsened because they are all forced to follow the same rules mechanically. Governments also gain experience over time. It's possible for governments to craft more specific and unique policies. Environmental pollution is effectively reduced, and the government's investment improves efficiency in pollution control projects. ER can only be implemented if the general public is aware of protecting the environment. Companies' environmental behavior cannot be effectively restrained if the general public does not participate in environmental protection. As public involvement rises, businesses will feel compelled to establish environmental management standard systems independently.

It is clear from the empirical research findings that the effect of environmental regulations on health expenditures varies significantly from one region to the next. In terms of health expenditure, ecological regulations for the median health expenditure have a negative relationship with health expenditure. While the industry consumes a large amount of high-polluting energy to promote economic growth, it is widely believed that this will have a negative effect on the health of residents and, consequently, on health expenditure (89). Because of this, CO_2_ emissions and healthcare expenditures should have a long-term relationship of co-integration.

TI has a negative relationship with carbon emissions, which is consistent with the findings of Amran et al. (90) and Khanfar et al. (91), who both fixed this negative relationship; however, The findings of Ganda et al. (92) are in direct opposition to this. When combating environmental degradation, technological innovation is a critical component. As a result, TI may be able to assist China in shifting its industrial and economic structure in the direction of more sustainable development.

### Effect of Regional Heterogeneity

Provincial heterogeneousness in environmental regulation and environmental quality may exist as a result of varying levels of development in different regions. Tobit random regression is carried out in three regions of China in this section. The outcomes are shown in Table 6 of our estimations. Environmental regulation has a substantial U-shaped influence on environmental quality (*p* = 1 and 5%, respectively). It's in line with the national sample's findings. Companies have a greater incentive to meet environmental standards when the food industry is subject to more stringent environmental regulations. CER's first-order coefficient is negatively impacted by ecological performance (93), while the second-order coefficient is somewhat positive but not statistically significant. In a nutshell, increasing the intensity of CER does not lead to an increase in inefficiency.

**Table 6:** Regional heterogeneity estimation results.

**Variable**	**Central**	**Eastern**	**Western**
ER	−77.01***	−3.15**	−8.53
	−19.58	−1.246	−6.607
Public Health	34,916	44.41**	3,843***
	−22,545	−20.29	−1,485
Control variables	YES	YES	YES
Constant	0.907***	0.928***	−0.0101
−0.149	−0.156	−0.0541
Wald test	107.84	81.34	111.41
	0	0	0
Rho	0.554	0.509	0.134
	−0.127	−0.133	−0.085
Log-likelihood	55.16	46.96	208.69
LR test	57.03	51.44	8.24
	0	0	0

1, 5, and 10% significance levels represents the ^***^, ^**^, and ^*^ respectively.

### Robustness Test

We employ the robust OLS approach to ensure the model's resilience. Of course, the OLS estimators' outputs were reliable (Table 7). The study's primary variables are scientific innovation and energy-environmental performance. We retest the robustness using a different dependent variable and a different mediating variable. An ECPI indicator is developed in this paper to measure the efficiency of a company's operations (94). The number of scientists employed by a company can also be used as a proxy for its innovation capacity. As a result (95), we enlist the aid of independent researchers to gauge the level of innovation within the company.

**Table 7:** Robust results OLS.

**Variables**	**(1)**	**(2)**
ER	−1.512***	−1.748***
HEXP	−0.1985***	−0.1054***
Innovation	−0.0505***	−0.082***
Structure	0.447***	0.356***
TO	0.088***	0.651***
CS	0.057***	0.064***
ES	−0.573***	−0.824***
Cons.	3.632***	3.435***

1, 5, and 10% significance levels represents the ^***^, ^**^, and ^*^ respectively.

**Figure 3:**
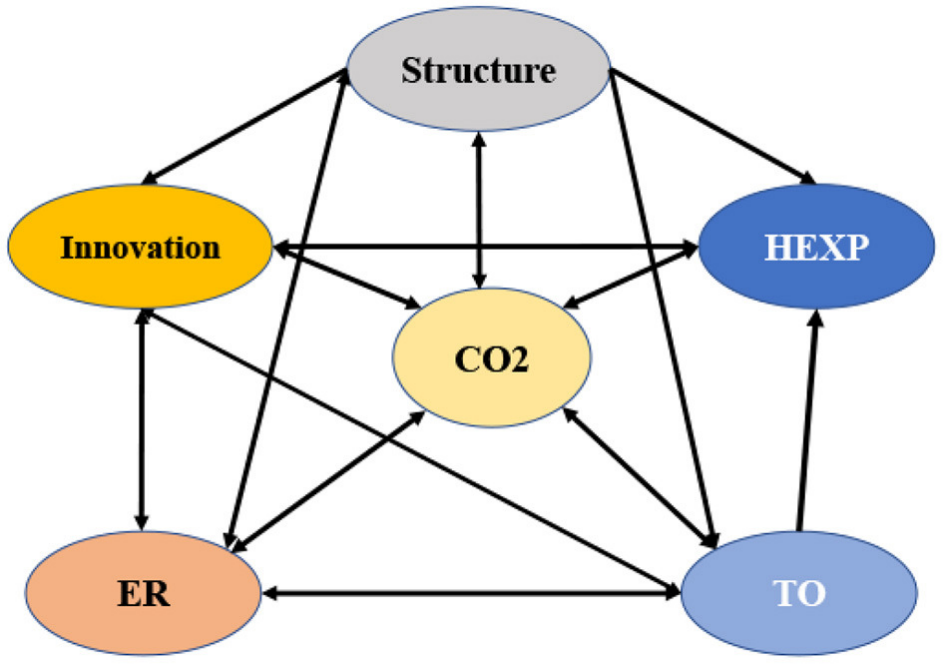
Pair-wise panel causality tests.

After examining the long-term relationship between variables, the Granger causality test will be used to determine whether or not there is a causal relationship between the variables. It is reasonable to expect a unidirectional or bidirectional causal relationship between the series if the variables are not stationary but after testing they are found to be cointegrated. Using an enhanced vector autoregressive (VAR) framework, the Granger causality hypothesis will be tested. Table 8 depicts the Granger causality between the variables over a short time interval. The environmental regulation Granger causes CO_2_ emissions level. The level of CO_2_ emissions Granger causes trade openness and a health expenditure. Furthermore, there is bidirectional relationships between trade openness and environmental regulation, trade openness and CO_2_ emissions. The arrow direction represents Granger causality (see Figure 3). This can be explained from the perspective of economic significance. As a result, environmental regulation is beneficial to the accumulation of innovation, and the strengthening of environmental regulation will result in the coordinated development of the environment and economy, as demonstrated by this finding.

**Table 8:** Pair-wise panel causality tests.

**Variable**	**CO_**2**_**	**ER**	**HEXP**	**Innovation**	**Structure**	**TO**
CO2	–	1.360**	0.136**	0.260***	0.407*	3.290**
	–	(0.002)	(0.528)	(0.451)	(0.378)	(0.042)
ER	0.177*	–	1.197**	2.922*	0.037***	0.580*
	(0.086)	–	(0.181)	(0.051)	(0.656)	(0.387)
HEXP	0.709**	4.335***	–	5.837	2.064*	0.391**
	(0.024)	(0.021)	–	(0.009)	(0.093)	(0.486)
Innovation	0.813*	0.867**	0.475*	–	0.119**	0.124*
	(0.251)	(0.240)	(0.351)	–	(0.554)	(0.675)
Structure	0.162**	4.353**	1.898**	0.898	–	3.578***
	(0.046)	(0.021)	(0.105)	(0.234)	–	(0.017)
TO	7.142*	4.322***	0.143*	3.571*	2.292**	–
	(0.001)	(0.038)	(0.660)	(0.017)	(0.078)	–

^***^, ^**^, and ^*^ represent 1, 5, and 10% significance levels, respectively.

## Conclusions and Policy Recommendations

### Conclusions

To ensure the long-term viability of the food industry, environmental regulations must be taken into consideration. Environmental quality and environmental law interact and influence one another in our study, which makes use of the Tobit model. The following are the results of our empirical investigation. At a definite level of regulatory intensity, this paper keeps up the Porter Premise in China's food industry. ER has a significant U-shaped association with the Food industry's energy-environmental performance, shown in the graph. Most province environmental regulation levels are currently below the tipping point. Achieving the Porter Premise will only be possible if China surpasses the modulation point in regulation intensity. Foreign direct investment and R&D investment have a substantial effect on energy-environmental performance, while the power structure has a negative impact.

To begin with, environmental regulations have a direct influence on the Food industry's energy-environmental performance, but technological innovation can also play a role in mediating this effect. Environmental law, energy-environmental performance, and scientific innovation play a role in this influence mechanism. The food industry's technological innovation is also affected by environmental regulation in a U-shape. If environmental regulations surpass specific intensity thresholds, the food industry may be forced to innovate and develop a competitive advantage.

Finally, the influence of environmental regulations on the environmental quality of the food industry in China is regionally distinct (96). ER form a U-shape relationship in the east, while the association between ER and environmental quality is negative. There is a substantial U-designed association between environmental quality and CER in the central region. Only when CER intensity exceeds 0.00404 can a win-win Porter outcome be attained. CER, MER, and environmental quality form a strong U-shape relationship in the western region.

### Policy Suggestions

According to the findings, environmental pollution can't be reduced by a low level of regulation. The ecological quality can only be improved if the environmental regulation intensity is increased beyond the inflection point. As a result, three types of environmental regulation must be strengthened (97). When it comes to controlling pollution, command-based regulation is a public approach.

To improve the quality of the environment, the management must spend more money on pollution control schemes. It is, however, a challenge for the government alone to achieve the desired results (98). Large businesses have a unique opportunity to reduce pollution by using market-based regulation tools. A fundamental shift from terminal management to source management should take place in how businesses use energy. In addition, citizens can raise their environmental awareness and become more involved in ecological monitoring. The government should swiftly enforce the law in response to citizen reports of environmental violations (47).

As a follow-up, the food industry should speed up the development of an innovation system to encourage industry modernization. Research shows that technical innovation has a good impact on environmental quality. Environmental technology research must be supported if the industry is to succeed. Investing more in green technologies will help companies better understand how to improve efficiency (99). Firms can respond to stringent regulations by supporting technological innovation. Soft technologies, such as high-tech talent and advanced management experience, must also be introduced by companies in addition to their clean, high-tech devices. The food industry's green development will benefit from companies' ability to digest and absorb technology, which will aid in the absorption and digestion of technology.

Environmental regulations have an impact on innovation activities, but it is important to distinguish between the effects of different regulations. In order to improve environmental quality, environmental regulations should be used effectively to encourage continuous technological innovation. The government should do more to promote technical advancements in information security. The government can increase subsidies for specific technologies to spur the practical impacts of environmental regulation. Aside from setting ecological standards for procurement, the administration can also remove unnecessary capacity in order to help the Food industry transition toward supply chain operations and green manufacturing.

Environmental regulations must be chosen in accordance with local conditions. Various one-size-fits-all environmental strategies in China do not adequately reflect the country's environmental management requirements and must be reformed immediately. The federal government should consider regional characteristics when formulating environmental regulation policies to make environmental regulations more widely applicable.

Researchers found that environmental regulations affect environmental quality differently in three regions. A shift to community-based and market-based environmental regulation is needed in China's eastern region. There is a need to reform resource and emission taxes in China to better leverage the market's regulatory character. It's also essential to raise awareness of environmental protection among the general population so that public oversight can be more effective (100). Increasing environmental pollution control projects and enforcing environmental laws are necessary for the central region. An increase in the level of command-based environmental regulation is needed over time. The government should use both command-based and market-based environmental regulations in the western part of the country. Using market-based processes such as emissions and payments permit interchange, companies can exert the externality effect of environmental regulations. It is difficult to meet environmental protection requirements in the western region because of the region's weak environmental carrying capacity and regressive fiscal development. Therefore, environmental enforcement should be stepped up to prevent the transfer of polluting productions and energy-inept from the east to the west (101). To sum it up, given the wide variations in environmental regulations across China's vast territory, the national government should exercise greater caution in enforcing environmental laws.

### Study Limitations and Future Research

The findings of this research are relevant to the food industry's green growth in the context of carbon impartiality. However, it needs to highlight some deficits in this research. It is impossible to evaluate environmental performance without taking into account CO_2_ emissions, primarily due to an absence of pollution emission data. No data on industrial sculpture, nitrogen oxide, or other pollutants is available to us, so the performance indicator does not take these into account. First of all, Zhou et al. (102) study does not have access to data from prefecture-level cities. Our study relied on a small sample of provincial data, and as a result, sample selection deviation is possible. The results derived from sample data are likely to be inaccurate and lacking in efficacy (103). The research, for example, could develop in three different directions in the future. Additional data on pollution emissions or prefectures at the city level will help us assess green industrial development. In order to conduct research on innovation and green productivity, we can use data from listed companies. As a result, the green movement will have a new outlook. The national carbon emission trading market was formally launched on July 16, 2021. To achieve carbon neutrality, this market-based policy tool will be critical. Using carbon emission trading to reduce industrial emissions and save energy is a fascinating and important topic. Researchers and policymakers will have a lot of work to improve the design of carbon emission trading mechanisms.

## Data Availability Statement

The raw data supporting the conclusions of this article will be made available by the authors, without undue reservation.

## Author Contributions

The author confirms being the sole contributor of this work and has approved it for publication.

## Conflict of Interest

The author declares that the research was conducted in the absence of any commercial or financial relationships that could be construed as a potential conflict of interest.

## Publisher's Note

All claims expressed in this article are solely those of the authors and do not necessarily represent those of their affiliated organizations, or those of the publisher, the editors and the reviewers. Any product that may be evaluated in this article, or claim that may be made by its manufacturer, is not guaranteed or endorsed by the publisher.
